# Multi-Level Analysis and Identification of Tumor Mutational Burden Genes across Cancer Types

**DOI:** 10.3390/genes13020365

**Published:** 2022-02-17

**Authors:** Shuangkuai Wang, Yuantao Tong, Hui Zong, Xuewen Xu, M. James C. Crabbe, Ying Wang, Xiaoyan Zhang

**Affiliations:** 1 School of Life Sciences and Technology, Tongji University, Shanghai 200092, China; wangshuangkuai@tongji.edu.cn (S.W.); 1731484@tongji.edu.cn (Y.T.); zonghui@tongji.edu.cn (H.Z.); 2 Department of Medicine Library, Tongji University Library, Tongji University, Shanghai 200092, China; 3 Department of Laboratory Medicine, Eastern Hepatobiliary Surgery Institute, Second Military Medical University, Shanghai 200438, China; xuewen-xu@163.com; 4 Wolfson College, Oxford University, Oxford OX2 6UD, UK; james.crabbe@wolfson.ox.ac.uk; 5 Institute of Biomedical and Environmental Science & Technology, University of Bedfordshire, Luton LU1 3JU, UK; 6 School of Life Sciences, Shanxi University, Taiyuan 030006, China

**Keywords:** tumor mutational burden, next-generation sequencing gene panel, omics data mining, tumor-infiltrating lymphocytes

## Abstract

Tumor mutational burden (TMB) is considered a potential biomarker for predicting the response and effect of immune checkpoint inhibitors (ICIs). However, there are still inconsistent standards of gene panels using next-generation sequencing and poor correlation between the TMB genes, immune cell infiltrating, and prognosis. We applied text-mining technology to construct specific TMB-associated gene panels cross various cancer types. As a case exploration, Pearson’s correlation between TMB genes and immune cell infiltrating was further analyzed in colorectal cancer. We then performed LASSO Cox regression to construct a prognosis predictive model and calculated the risk score of each sample for receiver operating characteristic (ROC) analysis. The results showed that the assessment of TMB gene panels performed well with fewer than 500 genes, highly mutated genes, and the inclusion of synonymous mutations and immune regulatory and drug-target genes. Moreover, the analysis of TMB differentially expressed genes (DEGs) suggested that JAKMIP1 was strongly correlated with the gene expression level of CD8^+^ T cell markers in colorectal cancer. Additionally, the prognosis predictive model based on 19 TMB DEGs reached AUCs of 0.836, 0.818, and 0.787 in 1-, 3-, and 5-year OS models, respectively (C-index: 0.810). In summary, the gene panel performed well and TMB DEGs showed great potential value in immune cell infiltration and in predicting survival.

## 1. Introduction

Tumor mutational burden (TMB) is considered a potential biomarker for predicting the response and effect of immune checkpoint inhibitors (ICIs). TMB can characterize tumor genome stability and tumor microenvironment (TME) heterogeneity, and serve as the prevalent biomarker to predict cancer immunotherapy response [1]. A number of studies suggest that TMB was significantly different in responders and non-responders to ICIs in melanoma [2], non-small cell lung cancer [3,4,5], and colorectal cancer (CRC) [6,7], which then allows clinicians to determine who would benefit from immunotherapy.

Currently, TMB is determined using whole-genome sequencing (WGS) or whole-exome sequencing (WES), which limits routine clinical care due to high cost and time-consuming analysis [8]. In addition, some studies showed TMB can also be estimated from a panel of only a few hundred reliable genes. However, there are still two main controversies. One is that the optimal gene panel size varied [9]. The FDA have approved the Foundation One CDx (F1CDx) panel [10] and the FDA-authorized Memorial Sloan Kettering-Integrated Mutation Profiling of Actionable Cancer Targets (MSK-IMPACT) panel [7], which contain 315 and 468 cancer-related genes to assess TMB, respectively. Studies have shown that 150 genes can be used to assess TMB in non-small cell lung cancer (NSCLC) [11], and 170 genes in skin cutaneous melanoma (SKCM) and lung adenocarcinoma [12]. Campesato et al. [13] proposed that a comprehensive gene panel containing more than 300 cancer-related genes should be employed. Lyu et al. [14] used 24 genes to construct a TMB model as a predictor of cancer immunotherapy response. Given these studies, gene panels are mainly available for highly mutated cancers such as lung cancer, melanoma, colon cancer, and stomach cancer, and the accuracy may vary greatly in other cancers with less frequently mutated genes.

Another controversy is whether synonymous mutations should be calculated in TMB assessment. Most researchers only calculated TMB based on nonsynonymous mutations [15,16,17]. Some researchers thought that nonsynonymous mutations contributed more to the production of neoantigens on the tumor surface [18,19]. Others suggested that there was no clear evidence that synonymous mutations would be always silent and have no indirect contribution to the immunogenicity, so that different types of mutations are still included in the calculation of TMB assessment [20,21]. These controversies indicated that the effects of different mutation types should be evaluated in order to achieve accurate measurement of TMB.

To understand the underlying mechanism of TMB on predicting response in immunotherapy, several studies have indicated that high TMB tended to produce more neoantigens, leading to higher immunogenicity and better response to immunotherapy [22,23,24]. Moreover, a pan-cancer study based on the expression level of immune signature genes showed that differentially expressed immune signature genes in high- and low-TMB groups existed in a variety of cancers [25]. Besides, TMB was also correlated with immune cell infiltration and prognosis in distinct tumor types [26,27,28,29], which suggested that TMB may have an important impact on TME and prognosis. However, the relationship between the TMB genes, TME immune cell infiltration, and prognosis is not completely clear.

In this study, we comprehensively analyzed and evaluated the gene screening criteria of a TMB gene panel. Combined with the immune cell infiltration level, we investigated the association between TMB and cytokines in TME at the gene expression level. Furthermore, we constructed a predictive model for prognosis based on identified TMB genes (pictorial methodology in Figure S1).

## 2. Materials and Methods

### 2.1. Data Collection

Gene mutations and expression data of 32 different cancer types were downloaded from The Cancer Genome Atlas database (TCGA, http://gdac.broadinstitute.org, accessed on 4 March 2019). The dataset contained a total of 10,429 samples. The cancer types and the number of samples were listed in Table S1. We then compared our gene panel to the F1CDx (315 genes) gene panel and the MSK-IMPACT (468 genes) gene panel. The somatic mutations data annotated by muTect were downloaded. The RNA-seq Raw count, RNA-seq RSEM, and clinical information were obtained by the R package TCGAbiolinks. Gene IDs were collected from HGNC (https://www.genenames.org, accessed on 5 December 2018), and the CDS length was downloaded from the NCBI (ftp://ftp.ncbi.nlm.nih.gov, accessed on 5 December 2018). MHC affinity information was retrieved from Rooney et al. [30], TCIA [31], and TSNAdb [32].

### 2.2. Gene Source and Collection Method

PubTator [33] and GIDB [34] were utilized to extract co-occurrence genes in the literature (https://ftp.ncbi.nlm.nih.gov/pub/lu/PubTatorCentral/; http://bmtongji.cn/GIDB/index.html; Both were accessed on 22 April 2019). We downloaded the gene annotation files from these two databases. The gene ID was obtained from the HGNC database, and the cancer term was collected from MESH of NCBI.

The Gene Ontology (GO) entries with the word prefix “immune” in the annotation “Term” were used to select immune-related genes. We then downloaded the GO hierarchies file ‘go-basic.obo’ and human go annotation file ‘goa_human.gaf’ from the Gene Ontology database (http://geneontology.org, accessed on 25 March 2019).

NCCN (https://nycancer.com/nccn/, accessed on 25 October 2019) guidelines were used to obtain drug-target genes, diagnostic genes, and prognostic-related genes. Drug-target genes were obtained from the clinical trials database (https://clinicaltrials.gov, accessed on 25 October 2019).

Gene selection criteria in this paper are as follows:

1. The number of genes in the panel is below 500;
2. The gene mutation frequency is over 0.01;
3. Cancer-associated genes in the published literature are preferentially selected;
4. Immune regulatory genes are preferentially selected;
5. Drug-target genes, prognosis-related genes, or genes in clinical research are preferentially selected;
6. Synonymous mutations are included in the TMB assessment.

### 2.3. TMB Calculation

The “Variant_Classification” column of the MAF file from TCGA was selected for mutation frequency calculation and subsequent analysis. The mutation types included “Missense_Mutation”, “Frame_Shift_Del”, “Frame_Shift_Ins”, “In_Frame_Del”, “In_Frame_Ins”, “Nonstop_Mutation”, “Nonsense_Mutation”, “Splice_Site”, “Translation_Start_Site”, and “Silent”. In this study, the TMB value was calculated by the ratio of number of records in the “Hugo_Symbol” column to the total length of exons (38 million) [11] for each sample, and the gene length of cancer-specific gene panel (SepPanel) was the CDS length. After sorting by TMB values, the top 20% samples were considered to be the high-TMB group, and the bottom 20% samples were considered to be the low-TMB group [7]. TMB coefficient of determination (R-square, R^2^) was used to evaluate the correlation between SepPanel TMB and TMB level based on WES (WES TMB). The coefficient of determination is defined as the square of the correlation between predicted values (SepPanel TMB) and actual values (WES TMB); thus, it ranges from 0 to 1.

### 2.4. DEGs and Functional Enrichment Analysis

The R package limma was utilized to obtain DEGs with adjusted *p* < 0.05 and |logFC| > 1 as the cut-off among the high- and low-TMB groups. ClusterProfiler was used for functional enrichment analysis. The STRING (http://www.string-db.org, accessed on 25 February 2020) database was used to construct a PPI network. Cytoscape software was used to visualize the modules network obtained by MCODE [35]. For correlation analysis, expression values of zero in samples were regarded as outliers and removed [36].

### 2.5. TMB Differentially Expressed Genes and Immune Cell Infiltration Process

TIMER [37] (http://cistrome.dfci.harvard.edu/TIMER/, accessed on 7 March 2019) and CIBERSORT [38] (https://cibersort.stanford.edu/, accessed on 16 May 2019) were used to calculate the immune cell infiltration score. We used the Wilcox test to analyze CD8^+^ T cell infiltration levels between high- and low-TMB groups (*p* < 0.05). Pearson correlation analysis was used to screen the DEGs and cell infiltration marker genes (*p* < 0.05).

### 2.6. Prognosis Prediction Model Construction

Survival analysis was performed using the R package “Survival”. Kaplan–Meier was used to calculate survival rate, and the log-rank test was used to test the difference between survival curves. The R package “glmnet” was used to perform LASSO Cox regression model analysis. In LASSO model construction, 10-fold cross-validation was used to find the optimal value of the penalty parameter λ, and then the prognostic genes with regression coefficients were selected based on the optimal λ value. The risk score of each patient was calculated via the non-zero coefficient in Cox regression analysis. The R package “RMS” was used to draw nomograms and calibration curves. The receiver operating characteristic curve (ROC) and Harrell consistency index (C-index) were used to evaluate the model performance.

The risk score formula was as follows, in which “expr_genen_” represents the expression level of non-zero coefficient gene in Cox regression analysis. Coefficient represents the non-zero coefficient in Cox regression analysis.

Risk score = expr_gene1_ × coefficient + expr_gene2_ × coefficient +…+ expr_genen_ × coefficient (1)

## 3. Results

### 3.1. Establishment of Screening Criteria for TMB Gene Panel Collection

**Figure 1:**
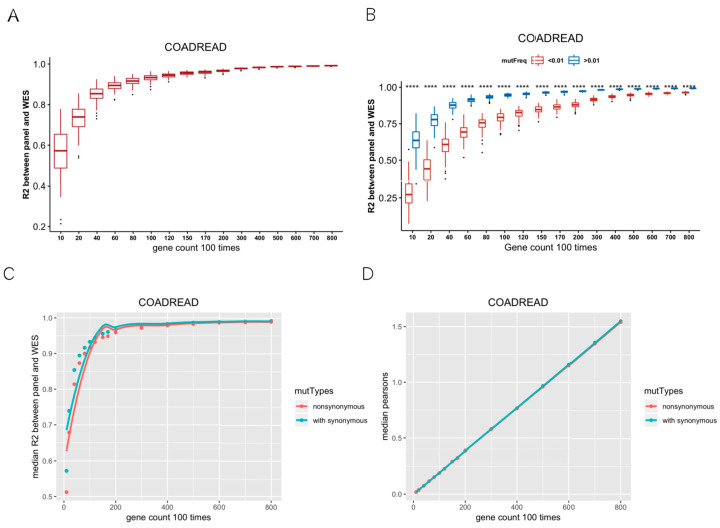
Establishment of screening criteria for TMB gene panel collection. (**A**) Coefficient of determination (R^2^) between random gene panel TMB from mutated gene and WES TMB. With the increase in random gene in gene panel, R^2^ had a tendency of becoming flat. (**B**) R^2^ between random gene panel TMB from high-mutation group (mutation frequency > 0.01) and WES TMB (blue boxplot), low-mutation group (mutation frequency < 0.01), and WES TMB (red boxplot). R^2^ values were significantly different (‘****’ means Wilcox test *p* value < 0.0001) between these two groups. (**C**) Median R^2^ between synonymous mutations included and excluded for gene panel TMB and WES TMB. (**D**) Median gene length of synonymous mutations included and excluded from random gene panel.

We calculated TMB based on WES across 32 cancer types. TMB varies among different cancers, among which SKCM showed the highest median TMB. In CRC, STAD, UCEC, and SKCM, samples with TMB greater than 20 muts/MB were more than 5% (Figure S2). Firstly, we randomly selected 10, 20, 40, 60, 80, 100, 120, 150, 170, 200, 300, 400, 500, 600, 700, 800 genes, repeated 100 times per gene number, and then calculated TMB per gene panel. R^2^ values indicated the correlations between the random gene panel TMB and WES TMB. In Figure 1A, R^2^ values of the curve rose and gradually tended to become asymptotic as gene numbers increased, which indicated that it is feasible to use hundreds of genes (<500) to predict the WES TMB (Figure 1A). Besides, the optimal number of genes also varied in different cancer types. For instance, the curve was not flattening as gene numbers increased in PCPG, TGCT, and KIRP (Figure S3). Secondly, we divided the mutant genes into two groups with mutation frequency of 0.01 as a cut-off value. We randomly selected 10–800 genes from the mutated genes, repeated 100 times, and calculated R^2^ between the random gene panel TMB and WES TMB. The R^2^ values of the random gene panel were higher at mutation frequency > 0.01, compared to at mutation frequency < 0.01 (Figure 1B). Therefore, we selected genes with mutation frequency over 0.01 to measure TMB. Thirdly, the median R^2^ of random gene panel TMB and WES TMB with or without synonymous mutations was compared. The R^2^ containing synonymous mutations was higher than that only calculated with non-synonymous mutations (Figure 1C; for other cancer types, please see Figure S4), and the sizes of corresponding gene sets were similar (Figure 1D). Therefore, we included synonymous mutations when calculating TMB. According to the above criteria, 32 TMB SepPanels were obtained, with a total of 2144 genes (Table S2).

### 3.2. Comprehensive Analysis of SepPanel in R^2^, GO Enrichment, and Mutation Frequency

**Figure 2:**
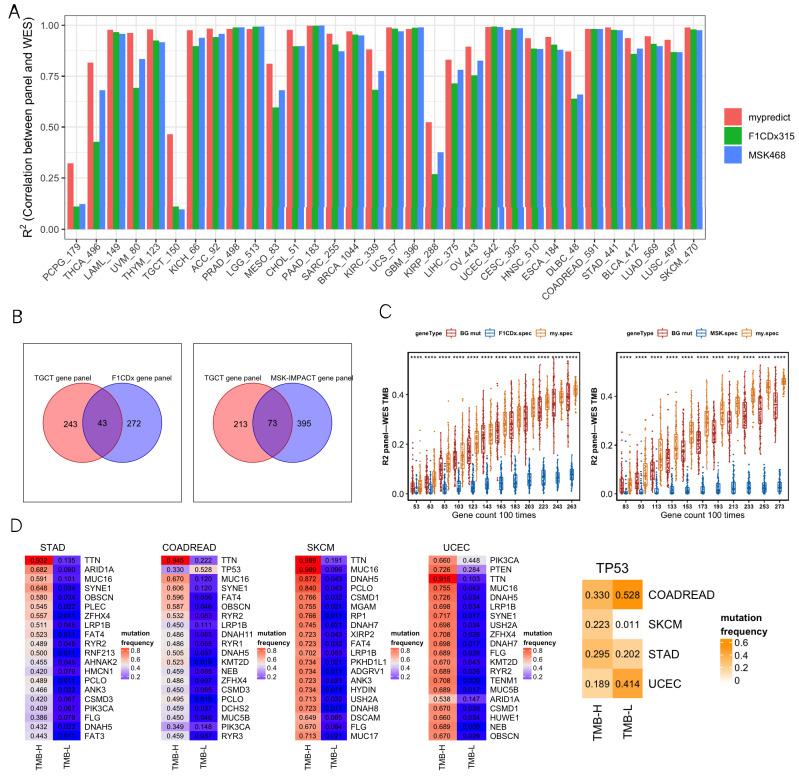
(**A**) R^2^ between gene panel TMB and WES TMB across 32 cancer types. SepPanel (red bar), F1CDx (green bar), and MSK (blue bar). (**B**) Venn plots of TGCT-SepPanel with F1CDx (left), TGCT-SepPanel with MSK (right) gene sets. (**C**) The boxplot on the left panel represents R^2^ between gene panel TMB and WES TMB across TGCT-SepPanel (orange boxplot), random gene panel (red boxplot), and F1CDx gene panel (blue boxplot). The boxplot on the right panel represents R^2^ between gene panel TMB and WES TMB across TGCT-SepPanel (orange boxplot), random gene panel (red boxplot), and MSK gene panel (blue boxplot). ‘****’ means Wilcox test *p* value < 0.0001. The dots out of box are outliers. Q1 and Q3 represent top and bottom quantile of data batch. Lower limit is Q1-1.5(Q3-Q1). Upper limit is Q3+1.5(Q3-Q1). Outliers are the data out of range (lower limit to upper limit). (**D**) The four heatmaps on the left panel represent the top 20 differentially mutated genes among high- and low-TMB group in STAD, COADREAD, SKCM, and UCEC. The heatmap on the far right represents TP53 mutation frequency of high- and low-TMB group in these four cancer types. The numbers in the heatmap represent mutation frequencies in these groups.

We comprehensively analyzed R^2^, GO enrichment, F1CDx/MSK, and differentially mutated genes. For most cancer types, most R^2^ values were close between the SepPanel, F1CDx, and MSK (all above 0.8, Figure 2A). Especially, it increased by 8% in THCA, UVM, DLBC, MESO, KIRC, and OV. More interestingly, in PCPG, TGCT, and KIRP, the R^2^ of SepPanel TMB and WES TMB increased by 30% compared to it in F1CDx/MSK.

GO enrichment analysis was then performed to explore the gene functional difference for greatly increasing R^2^ in TGCT SepPanel compared to F1CDx and MSK. There were 243 unique genes in the TGCT SepPanel and 272 unique genes in F1CDx (Figure 2B). The TGCT-SepPanel 243 genes were enriched in regulation of immune response. The F1CDx 272 genes were enriched in cell proliferation and differentiation. Meanwhile, there were 213 unique genes in the TGCT-SepPanel, and 395 unique genes in the MSK (Figure 2B). The TGCT-SepPanel 213 genes were also enriched in regulation of immune response. The MSK 395 genes were enriched in cell proliferation and differentiation, and regulation of immune response. The random gene set from the SepPanel, F1CDx/MSK, and all mutant genes were further analyzed. We used 43 common genes between SepPanel and F1CDx and 73 common genes between SepPanel and MSK as the basic gene set, then combined with randomly selected genes from SepPanel, F1CDx, MSK, or all mutant genes. With increasing gene number, R^2^ in TGCT-SepPanel TMB and WES increased, and TMB R^2^ was significantly greater than that in F1CDx/MSK (Figure 2C).

To further understand the mutation frequency of more than 5% of all genes in the high- and low-TMB groups, Fisher’s test was used to determine whether there was a significant difference in mutation frequency for genes between the two TMB groups. The most common mutant genes with significant differences (FDR < 0.05) in high- and low-TMB groups were found in CRC, STAD, SKCM, and UCEC. Figure 2D shows that the top 20 (ranked by mutation frequency) differentially mutated genes overlapped in the above four cancer types. Among them, TTN, MUC16, and LRP1B were highly mutated in all four cancers. Most genes seemed to preferentially mutate in the high-TMB group. Interestingly, we found that TP53 tended to preferentially mutate in the low-TMB group in CRC and UCEC. To further identify the genes that can potentially generate neoantigens in the SepPanel of CRC, STAD, SKCM, and UCEC, genes with high expression level, high mutation frequency, and high MHC affinity were analyzed. Finally, we screened 4, 14, and 12 genes in the SepPanel of CRC, STAD, and UCEC that may derive neoantigens (Table S3).

### 3.3. TMB-Related Differentially Expressed Genes

**Figure 3:**
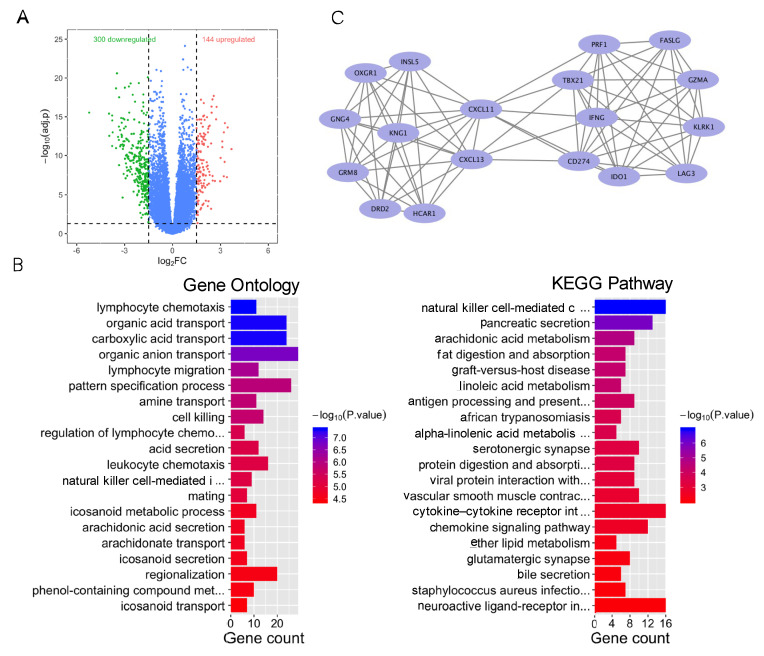
Bioinformatics analysis of differentially expressed genes of the high- and low-TMB group. (**A**) Volcano plot of differentially expressed genes among the high- and low-TMB group. (**B**) GO and KEGG enrichment of differentially expressed genes in the high- and low-TMB group. Blue color indicates lower *p* value. Red color indicates higher *p* value. (**C**) Main gene clusters.

As a case exploration, colorectal cancer patients were divided into a high-TMB group (the top 20% of TMB) and a low-TMB group (the bottom 20% of TMB). We identified a total of 444 differentially expressed genes (*p* < 0.05 and |logFC| > 1), including 144 upregulated genes and 300 downregulated genes (Figure 3A). GO enrichment analysis showed that DEGs were associated with immune regulation, including lymphocyte chemotaxis. KEGG pathway analysis found that DEGs were enriched in immune regulation and substance transport in the digestive system and lipid metabolism (Figure 3B), including natural killer cell-mediated cytotoxicity and pancreatic acid secretion. These results suggested that immune regulation plays an important role in the TMB group.

In addition, the most significant gene module (module 1), with a score of 9.412, was calculated via MCODE (Figure 3C). Module 1 was composed of 18 nodes and 80 edges. GO enrichment analysis demonstrated that those genes in module 1 were enriched in immune-related ontologies, including regulation of cell–cell adhesion and positive regulation of cell killing.

### 3.4. Analysis of TMB-Related DEGs and CD8^+^ T Cell Infiltration

**Figure 4:**
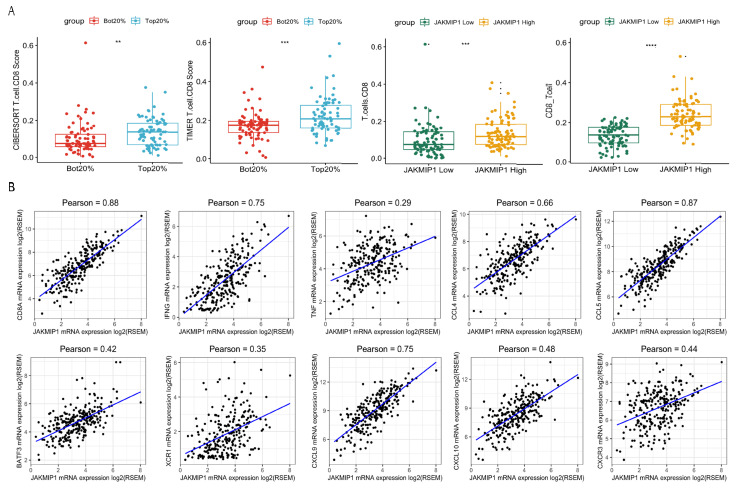
Differentially expressed genes of the high- and low-TMB group related to CD8^+^ T cell infiltration. (**A**) CD8^+^ T cell infiltration score of the high- and low-TMB group (left two plots). CD8^+^ T cell infiltration score of JAKMIP1 high- and low-TMB group (right two plots). ‘**’ means Wilcox test *p* value < 0.01. ‘***’ means Wilcox test *p* value < 0.001. ‘****’ means Wilcox test *p* value < 0.0001. (**B**) Pearson correlation of JAKMIP1 expression with CD8A, IFNG, TNF, CCL4, CCL5, BATF3, XCR1, CXCL9, CXCL10, and CXCR3. Any sample with JAKMIP1, CD8A, IFNG, TNF, CCL4, CCL5, BATF3, XCR1, CXCL9, CXCL10, and CXCR3 expression of 0 was removed.

We then analyzed the infiltration levels of B cells, CD4 T cells, CD8^+^ T cells, neutrophils, macrophages, and dendritic cells in colorectal cancer. The immune cell infiltration score was obtained via “CIBERSORT” and “TIMER”. The Wilcox test revealed that infiltration levels of CD8^+^ T cells were significantly different between high- and low-TMB groups (Figure 4A). To investigate the key genes, the Wilcox test was used to determine which genes were associated with infiltration levels. We found that Janus Kinase and Microtubule Interacting Protein 1 (JAKMIP1) expression was significantly correlated with CD8^+^ T cell infiltration levels (Figure 4A). In order to further understand whether the JAKMIP1 gene was involved in the process of CD8^+^ T cell infiltration, we used Pearson correlation analysis to evaluate gene expression between JAKMIP1 and several cell marker genes. In particular, JAKMIP1 significantly correlated with the expression levels of markers of CD8^+^ T cells CD8A, IFNG, and TNF (Figure 4B, *p* < 0.001), whereas, there was no correlation with other members of the JAK family (*p* > 0.05).

Subsequently, we explored the association of gene expression between JAKMIP1 and other chemokines related to IFN-γ signaling, and leukocyte activation and migration. Among these genes, CCL4, CCL5, CXCL9, CXCL10, and CXCR3 were significantly associated with the gene expression levels of JAKMIP1 (Figure 4B, *p* < 0.001).

The result showed strong correlations between JAKMIP1 and chemokine gene expression level. This suggested that JAKMIP1 may not regulate CD8^+^ T cell infiltration through microtubule interaction like other JAK family members, but through leukocyte migration, DCs, and T-cell recruitment. 

### 3.5. Prognostic-Related Genes of TMB-Differentially Expressed Genes

**Figure 5:**
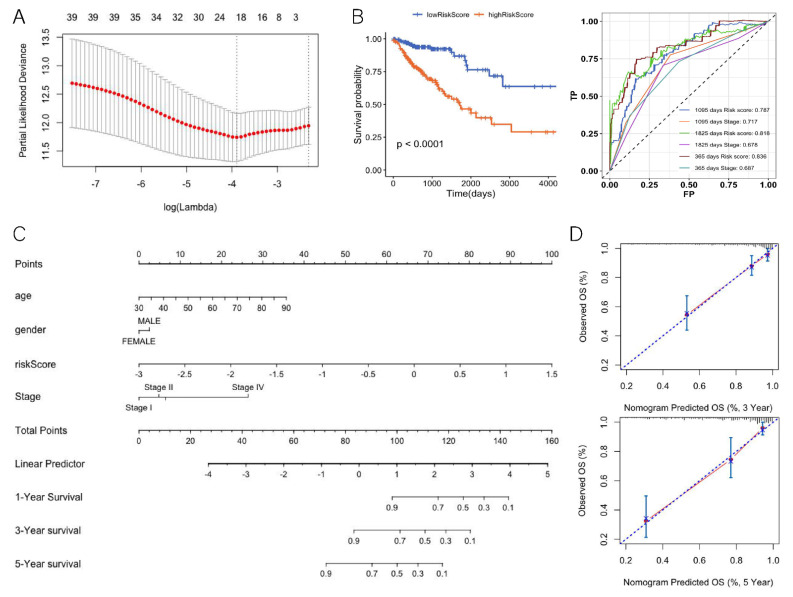
Differentially expressed genes of the TMB high and low groups related to prognosis. (**A**) LASSO cox regression feature-selection results. (**B**) Risk score prognostic curve (left) and risk score ROC curve (right). (**C**) Nomogram for predicting the 3- and 5-year OS. (**D**) Calibration plot for nomogram-predicted and observed 3- and 5- year survival rates.

We investigated the potential relevance of DEGs and prognosis. There were 39 DEGs significantly associated with overall survival (OS) according to Cox regression analysis. We performed LASSO Cox regression for these 39 gene-expression values. To avoid over-fitting, we used 10-fold cross validation to select the model (Figure 5A). The LASSO Cox regression showed that the coefficients of 19 genes were over 0 and the risk scores for patients were also calculated (Table 1, Equation (1)). According to the median risk score 0.46, the patients were divided into a high-risk group and a low-risk group (hazard ratio: 4.006; 95% CI: 2.331–6.885; *p* < 0.0001) (Figure 5B). The ROC analysis of time dependence showed that the AUCs of the 19 genes modeled were 0.836 (1 year), 0.818 (3 years), and 0.787 (5 years), respectively (Figure 5B). To understand whether the risk score could be used as an independent prognostic factor, multivariate Cox regression analysis was performed on age, tumor stage, and gender (Table 2). The results showed that the risk score was still significantly correlated with the prognosis when considering other clinical factors. Those factors found to be independently predictive of patient survival outcomes in the multivariate analyses were selected to construct the nomogram (Figure 5C). With respect to the nomogram of OS, the C-index between the predicted OS and the observed OS was 0.81 (*p* < 0.001). Moreover, the calibration curves indicated a good consistency between observed and predicted values for 3- and 5-year OS (Figure 5D).

## 4. Discussion

The TMB determined by WES has been established as a predictive biomarker of response to immunotherapy in several cancers. The time and cost still limit the application of WES TMB assessment in clinical situations. A gene panel for targeted sequencing is proposed to be an alternative to WES. We explored the criteria of gene panel selection for assessing TMB including gene number, mutation frequency, immune-related effects, and drug-target genes, and finally constructed 32 Tumor SepPanels. GO enrichment and random gene panels were further analyzed for TGCT (the highest improvement in R^2^). We then investigated the DEGs among the high- and low-TMB group. The JAKMIP1 gene was identified and that may play an important role in immune regulation in high-TMB groups. Prognosis analysis also indicated that TMB-related DEGs could become potential markers for predicting OS.

The performance of R^2^ in SepPanels was compared in F1CDx and MSK. For most cancers, R^2^ values were relatively high. However, in PCPG, TGCT and KIRP, the R^2^ values were all small in SepPanel, F1CDx, and MSK. Firstly, the factors affecting the regression coefficient may lower mutation amounts in these tumors. TMB was both low in PCPG and TGCT, and the highest TMB was only close to 1 mut/MB. However, TMB was high in KIRP (Figure S2), with most samples greater than 1 mut/MB. KIRP TMB ranked middle in 32 cancers. Secondly, R^2^ rose with increasing gene numbers, but the curve had no tendency to become flat (Figures S3 and S4). Moreover, for the SepPanels of PCPG, TGCT, and KIRP, we added other cancer types of driver genes and genes with high mutation frequency in the SepPanel. We found that the R^2^ increased, but still ranged from 0.45 to 0.55. Besides, some factors including tumor-related genes, drug-target genes, immune genes, and gene mutation frequency were also considered in the selection of gene sets in this study; however, the results still showed no significant improvement in these three tumors. Previous studies reported that PCPG is a disease of endocrine and metabolic system disorders [39,40]. The calcium metabolism pathway was enriched in TGCT which usually shows microcalcification and may be caused by impaired calcium metabolism [41]. Peroxisome and aldosterone synthesis and secretion pathways were enriched in KIRP, and the development of KIRP is related to the lack of peroxisomes, which can be used for the treatment of metabolism [42,43]. Therefore, we tried to include new genes such as metabolic pathways including glycolysis, gluconeogenesis, and carbohydrate digestion and absorption in PCPG SepPanels. The R^2^ reached 0.65 in PCPG, TGCT, and KIRP. This demonstrated that function enrichment results and cancer specific pathways may need to be particularly included in the gene panels of these three cancers.

Interestingly, the R^2^ of the TGCT-SepPanel significantly improved compared to F1CDx and MSK. It showed the specificity of TGCT compared with other solid tumors in TMB. TGCT has a special sensitivity to cisplatin-based chemotherapy, but 15–20% of patients who relapse on first-line or second-line treatment usually perform poorly and even require third-line treatment [40]. These patients may benefit from therapy with ICIs [41]. Some studies had shown that the PD-1 inhibitor antibody therapeutic drug Nivolumab had a positive effect in the treatment of TGCT [42,43]. This illustrates the advantages of our SepPanel method compared to these two classical gene sets.

It is known that some driver mutations might be potentially used to evaluate the efficacy of ICI monotherapy [44]. We counted driver mutation genes in SepPanel. There are 897 driver genes in the total 2144 genes of TMB SepPanel across 32 cancer types (41.8%). Driver genes accounted for more than 50% in most SepPanels (Figure S5). Genes with high expression, high mutation frequency, and high MHC affinity were selected in CRC, STAD, UCEC, and SKCM for the proportion of samples where more than 5% of TMB were greater than 20 muts/MB. In the gene list (Table S3), BRCA1/BRCA2 were reported and may promote neoepitope formation in high-grade serous ovarian cancer [45]. MUC16 neoantigenic clones involved selective loss in metastatic progression [46]. MUC4 is a novel tumor antigen for pancreatic cancer immunotherapy [47]. It is suggested that the potential neoantigen can be predicted from the SepPanel.

In colorectal cancer, we found strong correlations between JAKMIP1 and chemokine gene expression levels. This suggests that JAKMIP1 gene expression may regulate chemokine migration to boost the immune circulation in TME. BATF3- and CXCR1-DCs secrete CD141, which primes CD8^+^ T cells for anti-tumor activity and generates CXCL10 to recruit specific CD8^+^ T cells [48,49,50]. CCL4 and CCL5 stimulate the expression of CCR5 in DCs to migrate into tumors [51,52]. Therefore, the JAKMIP1 gene might induce the secretion of chemokines CCL4 and CCL5, which promote the recruitment of DCs into tumors. Then, DCs secrete chemokines CXCL9, CXCL10, and CXCR3 to induce T-cell infiltration. Thus, we suggest that the TMB-related gene JAKMIP1 could enhance the immune activity of TME in colorectal cancer.

The TNM classification of malignant tumor staging methods cannot provide accurate information to clinicians for predicting patient survival time. Recently, several novel lncRNA-based [53] and immune gene-based models [54] have been reported, while another recent study showed a relatively higher TMB with limited benefit from EGFR/BRAF blockade in patients with microsatellite stable (MSS) and BRAF-mutated mCRC [55]. However, a TMB-related gene-based model was not studied. In this study, a 19-TMB-related-gene model was constructed for prognostic prediction in colorectal cancer. When stratified by important clinical factors, the model retained a strong prognostic ability. For high-dimension microarray data, overfitting is a high risk. Herein, we used the LASSO method to avoid the limitation of overfitting. Stratified analysis showed that the 19-TMB-related-gene model was independent of AJCC stage. The AUC and C-index reached 0.836 and 0.81, respectively.

This study still has some limitations. Firstly, with the updating of research literature and genes, it is critical to automatically renew specific gene panels. Secondly, this study lacks data on immunotherapy responses and validation of prognostic models. Thirdly, the process of immune cell infiltration is complex, and some oncogenic pathways of driving mutations [51] or virus infections may also influence the activation of the immune response [56]. Thus, experimental validation will be needed in future work [57,58].

## 5. Conclusions

In this study, we constructed 32 tumor SepPanels including genes with high mutation frequency, immune-related genes, drug-target genes, and genes in clinical research. SepPanel performed well on assessing TMB in different cancer types. Moreover, TMB DEGs were found to be correlated with immune cell infiltration and prognosis. This study demonstrated that the tumor SepPanel would help in clinical decision making for immunotherapy treatment, and TMB DEGs would be important to investigate for new immunotherapy targets and clinical prognostic evaluation.

## Figures and Tables

**Table 1:** LASSO Cox regression selected genes and coefficients.

Gene	Coefs	Gene	Coefs	Gene	Coefs	Gene	Coefs
FOXD1	0.030	TLE6	0.148	ACCN3	0.038	HOXD13	0.025
HOXC6	0.027	CATSPERB	−0.077	IGF2BP3	0.001	SFTA2	0.071
TNNT1	0.048	APC2	0.146	PTGER2	−0.100	ATOH1	−0.037
HOXC4	0.017	ISLR2	0.160	CBLN2	−0.131	TMEM61	−0.116
ANKRD43	−0.022	APOLD1	−0.091	CD38	−0.066		

**Table 2:** Multivariate logistic regression for predicting survival.

Characteristic	Hazard Ratio	*p*-Value
Risk Score		
Low Risk Score	1.00 (reference)	
High Risk Score	3.3807 (0.2958, 5.940)	<0.001
Age	1.0478 (0.9544, 1.071)	<0.001
Gender		
Female	1.00 (reference)	
Male	0.9657 (1.0355, 1.540)	0.884
Tumor Stage		
I	1.00 (reference)	
II	1.1566 (0.8646, 3.120)	0.774
III	1.8502 (0.5405, 4.919)	0.217
IV	5.4542 (0.1833, 14.791)	<0.001

## Data Availability

Data will be available on reasonable request.

## Supplementary Materials

The following supporting information can be downloaded at: https://www.mdpi.com/article/10.3390/genes13020365/s1, Figure S1: Methodology of gene panel selection and TMB gene analysis; Figure S2: TMB distribution in different cancer types; Figure S3: Coefficient of determination of linear regression (R2) between random gene panel TMB and WES TMB cross cancer; Figure S4: Median R2 between between synonymous mutations included and excluded for random gene panel TMB and WES TMB; Figure S5: Driver gene count in SepPanel cross 32 cancer types. Orange bar represents the gene count of SepPanel, blue bar represents overlap of SepPanel with driver genes; Table S1: Cancer types and the relative number of samples; Table S2: SepPanels in 32 cancer types; Table S3: Genes may derive neoantigens.
