# Supplementary material for: Multi-Level Analysis and Identification of Tumor Mutational Burden Genes across Cancer Types
Source: Genes (Basel). 2022 Feb 17;13(2):365. doi: 10.3390/genes13020365 (PMC8872466; doi:10.3390/genes13020365)
Supplement: Supplementary file 1 [file genes-13-00365-s001.zip › Supplementary_material.pdf]

Figure S1. Methodology of gene panel selection and TMB gene analysis. SepPanel selection criteria were conducted from gene number, mutation frequency and synonymous mutation included or not in calculating TMB. Gene collection were derived from literatures, immune related pathway, NCCN guideline and drug target gene. SepPanel were assessed from R^2, gene functional enrichment, random gene panel and potentially target gene. TMB related DEGs were analyzed the association with TILs and prognosis.

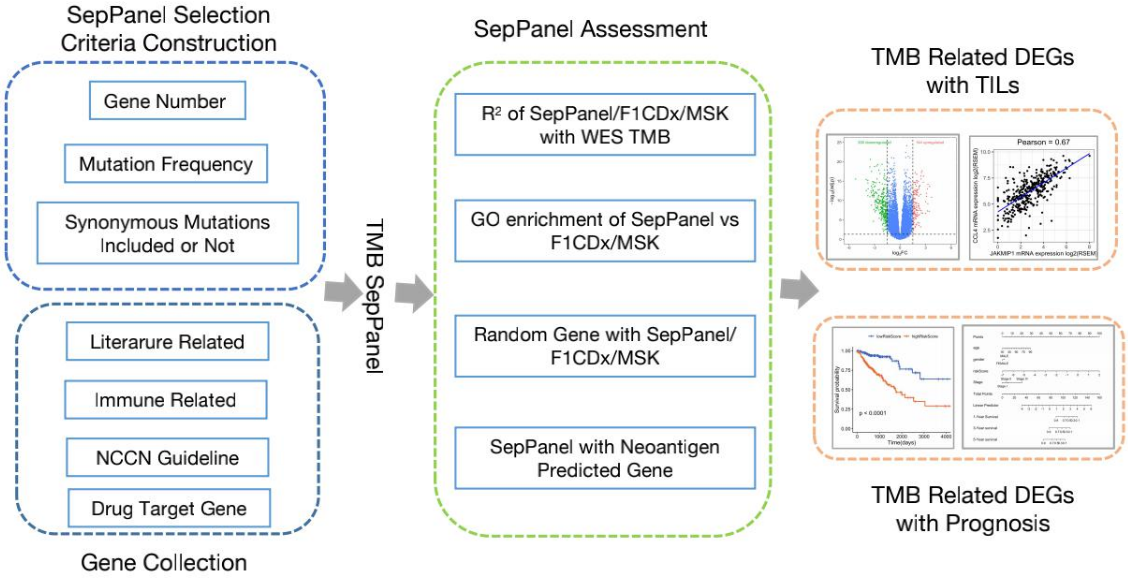

Figure S2. TMB distribution in different cancer types. The red dotted line represents the 20 muts/MB. Orange bar represents samples percent less than 20 muts/MB, and green bar represents samples percent more than 20 muts/MB.

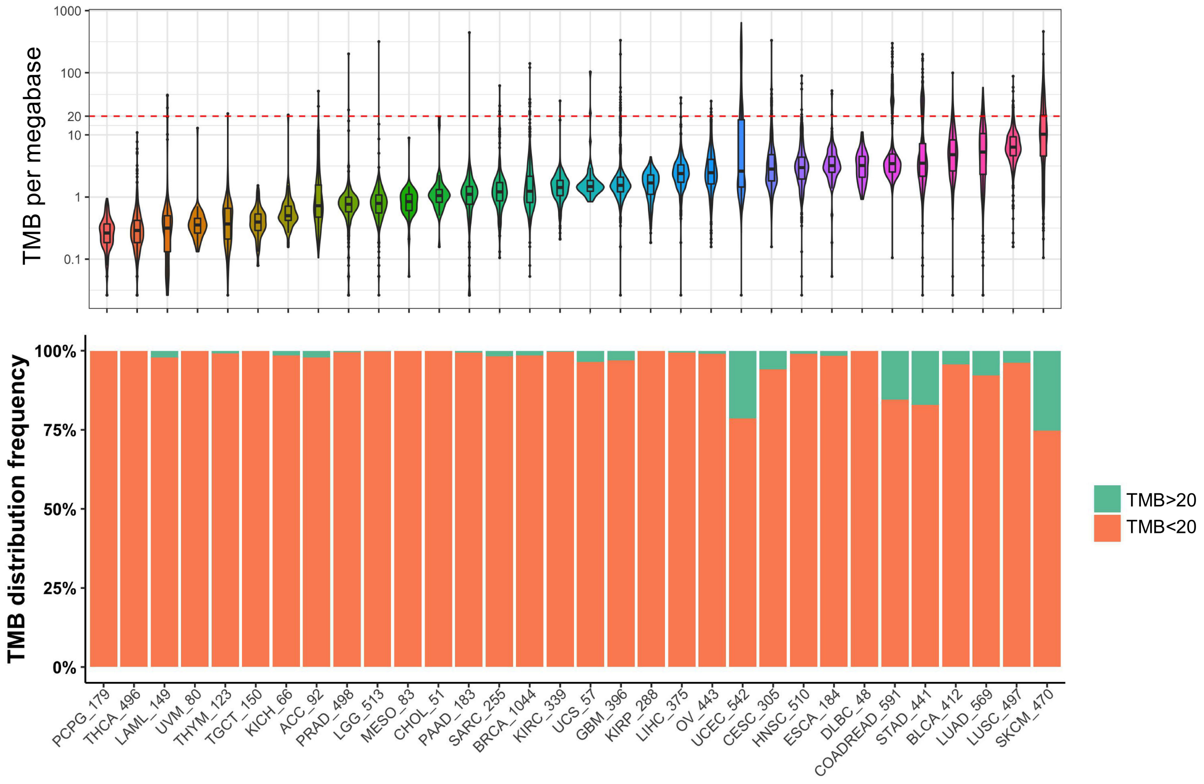

Figure S3. Coefficient of determination of linear regression (R^2) between random gene panel TMB and WES TMB cross cancer. With the increase of random gene in gene panel, R^2 had tendency becoming flat, except for KIRP, PCPG and TGCT.

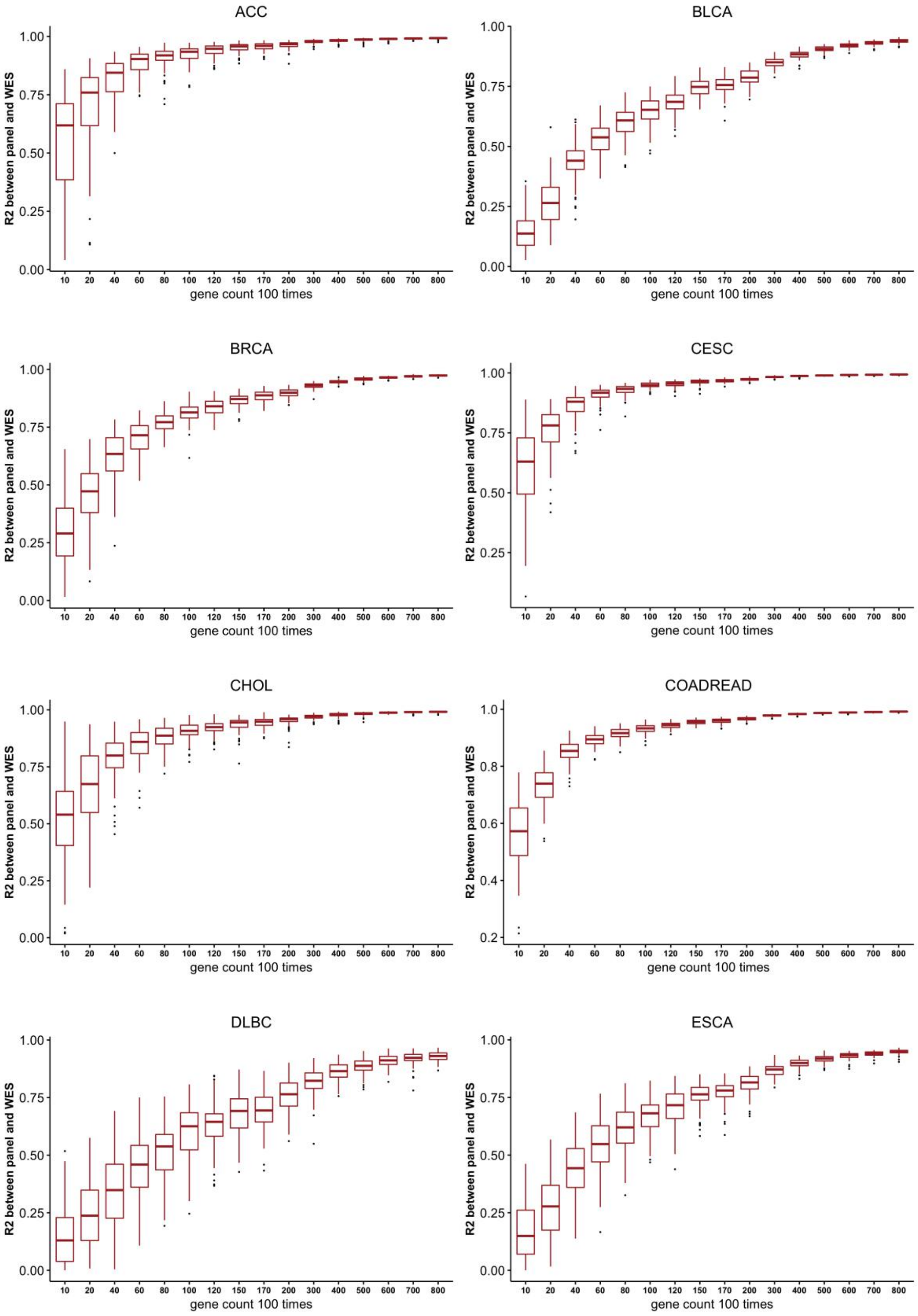

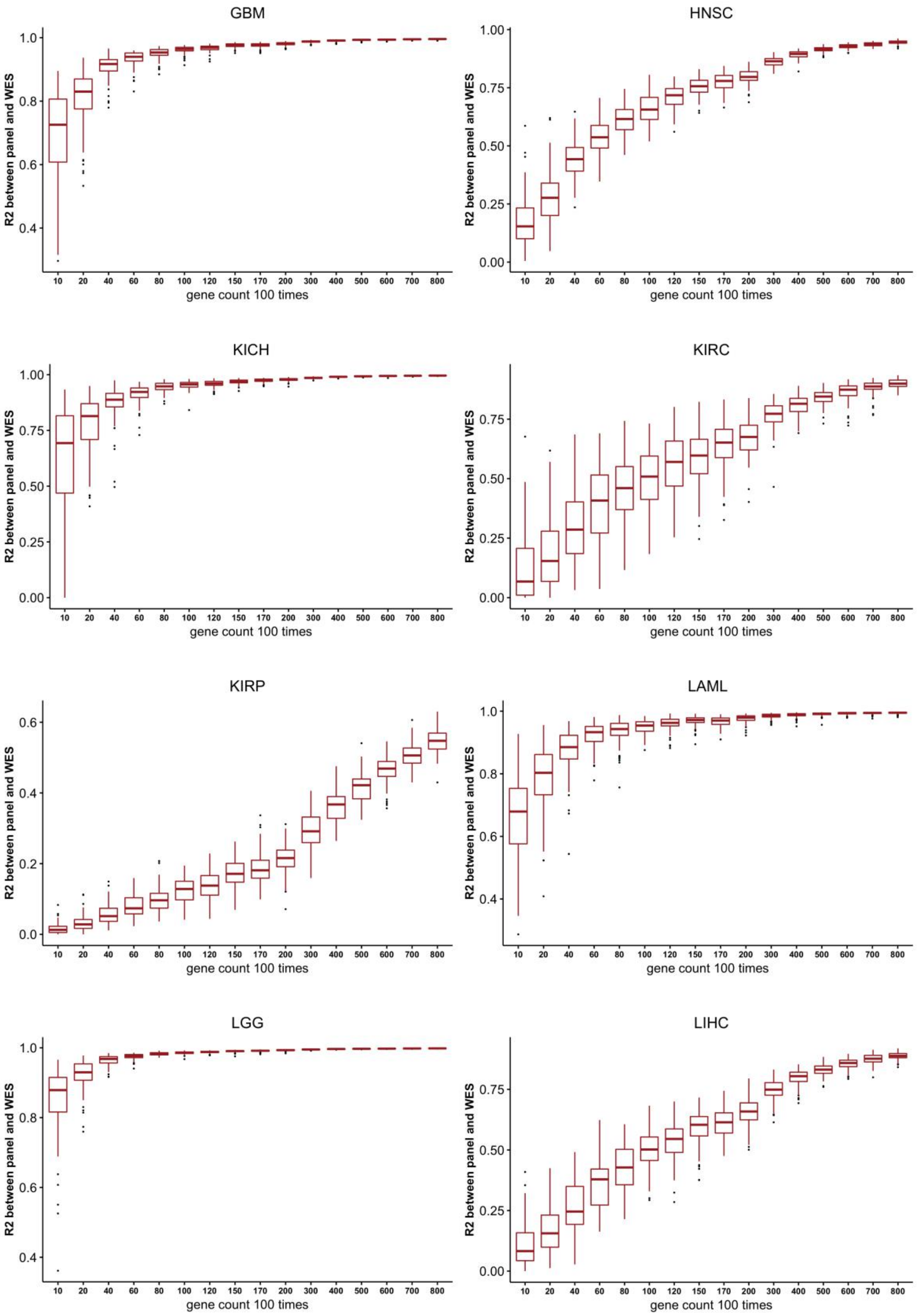
GBM
R2 between panel and WES
1.0
0.8
0.6
0.4
10 20 40 60 80 100 120 150 170 200 300 400 500 600 700 800
gene count 100 times
HNSC
R2 between panel and WES
1.00
0.75
0.50
0.25
0.00
10 20 40 60 80 100 120 150 170 200 300 400 500 600 700 800
gene count 100 times
KICH
R2 between panel and WES
1.00
0.75
0.50
0.25
0.00
10 20 40 60 80 100 120 150 170 200 300 400 500 600 700 800
gene count 100 times
KIRC
R2 between panel and WES
0.75
0.50
0.25
0.00
10 20 40 60 80 100 120 150 170 200 300 400 500 600 700 800
gene count 100 times
KIRP
R2 between panel and WES
0.6
0.4
0.2
0.0
10 20 40 60 80 100 120 150 170 200 300 400 500 600 700 800
gene count 100 times
LAML
R2 between panel and WES
1.0
0.8
0.6
0.4
10 20 40 60 80 100 120 150 170 200 300 400 500 600 700 800
gene count 100 times
LGG
R2 between panel and WES
1.0
0.8
0.6
0.4
10 20 40 60 80 100 120 150 170 200 300 400 500 600 700 800
gene count 100 times
LIHC
R2 between panel and WES
0.75
0.50
0.25
0.00
10 20 40 60 80 100 120 150 170 200 300 400 500 600 700 800
gene count 100 times

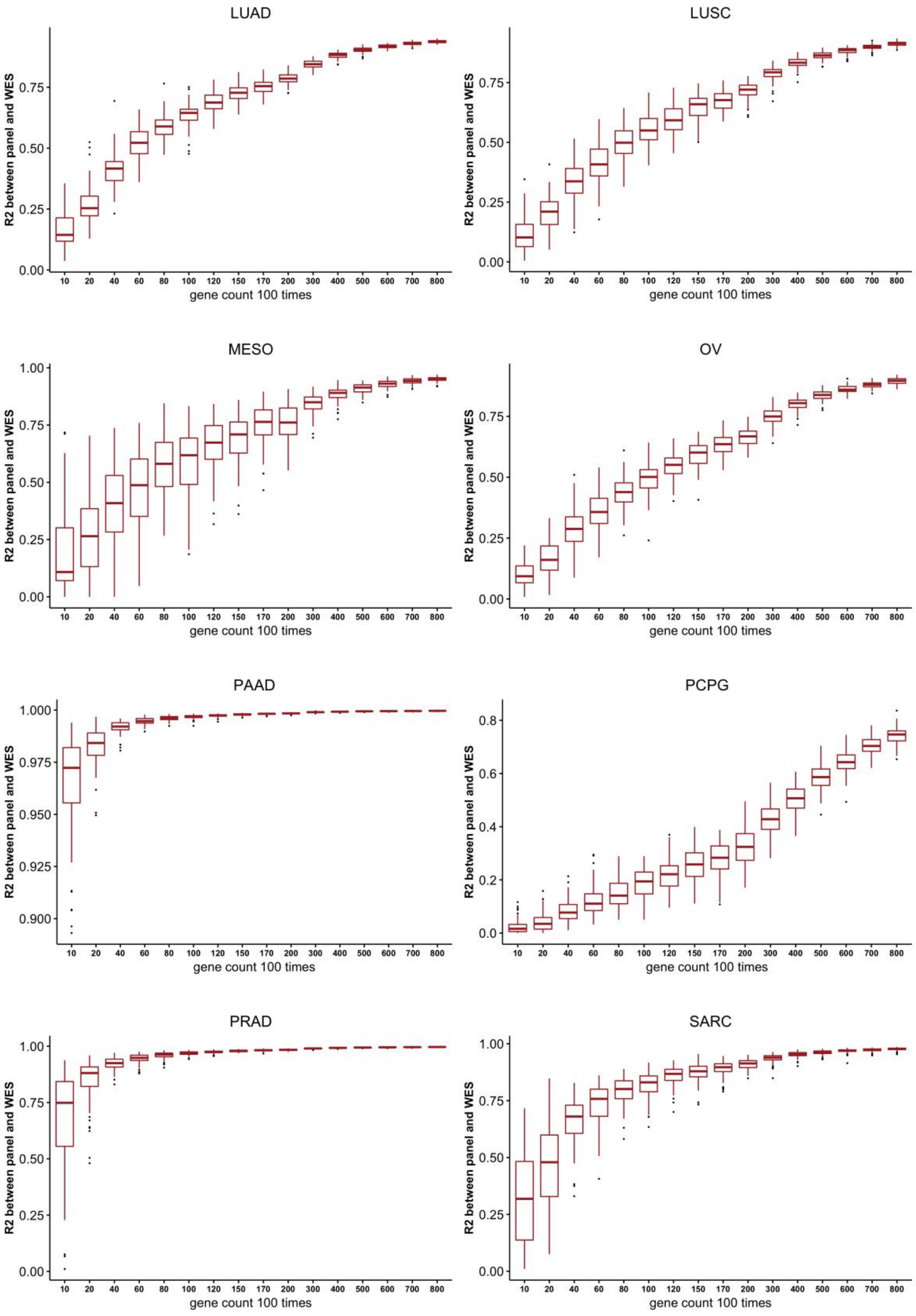

LUAD
LUSC
MESO
OV
PAAD
PCPG
PRAD
SARC
R2 between panel and WES
gene count 100 times
10 20 40 60 80 100 120 150 170 200 300 400 500 600 700 800
0.00 0.25 0.50 0.75 1.00
0.900 0.925 0.950 0.975 1.000
0.0 0.2 0.4 0.6 0.8

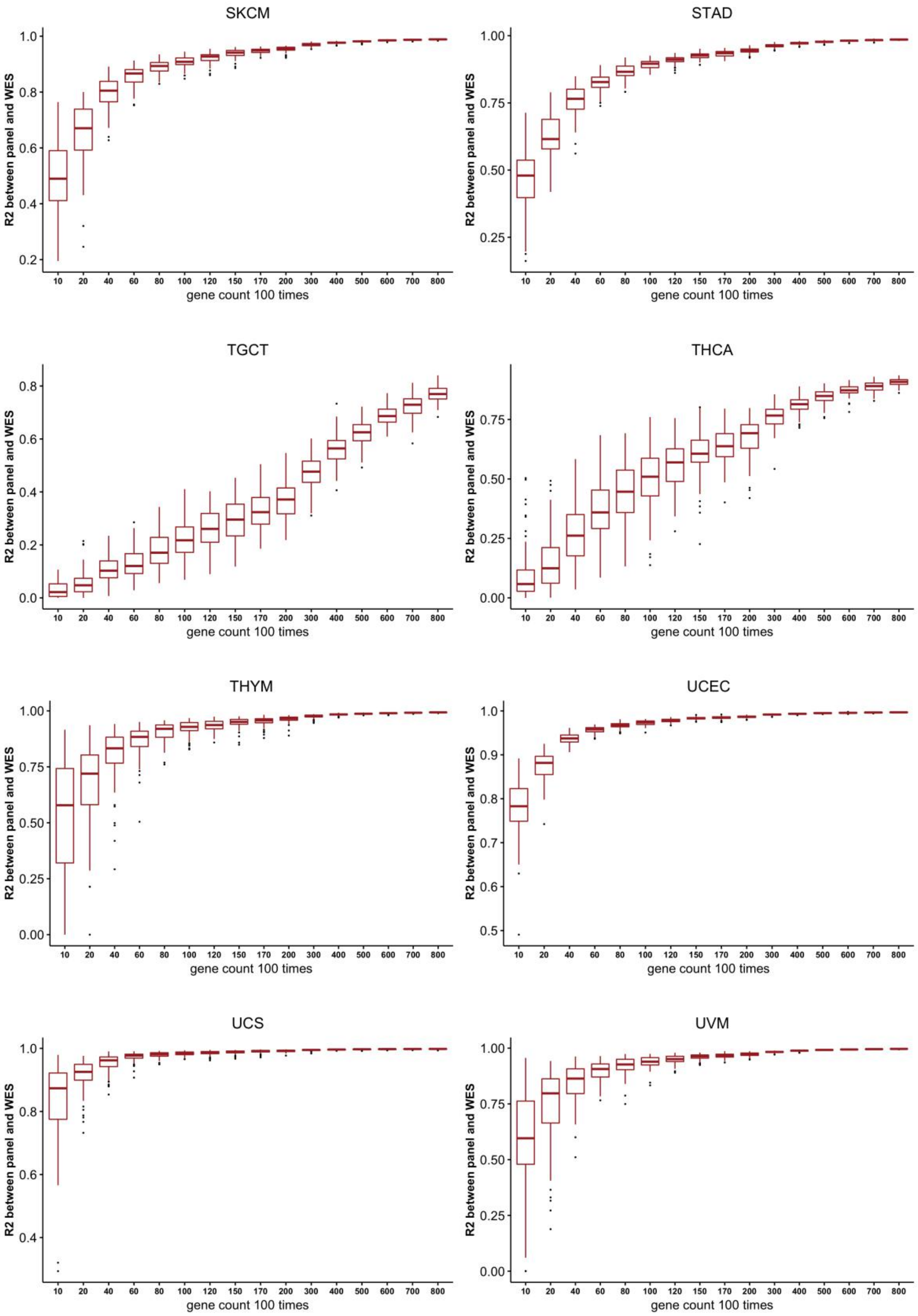

SKCM
STAD
TGCT
THCA
THYM
UCEC
UCS
UVM
R2 between panel and WES
gene count 100 times
10 20 40 60 80 100 120 150 170 200 300 400 500 600 700 800
0.2 0.4 0.6 0.8 1.0
0.25 0.50 0.75 1.00
0.0 0.2 0.4 0.6 0.8
0.00 0.25 0.50 0.75
0.00 0.25 0.50 0.75 1.00
0.5 0.6 0.7 0.8 0.9 1.0
0.4 0.6 0.8 1.0
0.00 0.25 0.50 0.75 1.00

Figure S4. Median R^2 between between synonymous mutations included and excluded for random gene panel TMB and WES TMB.

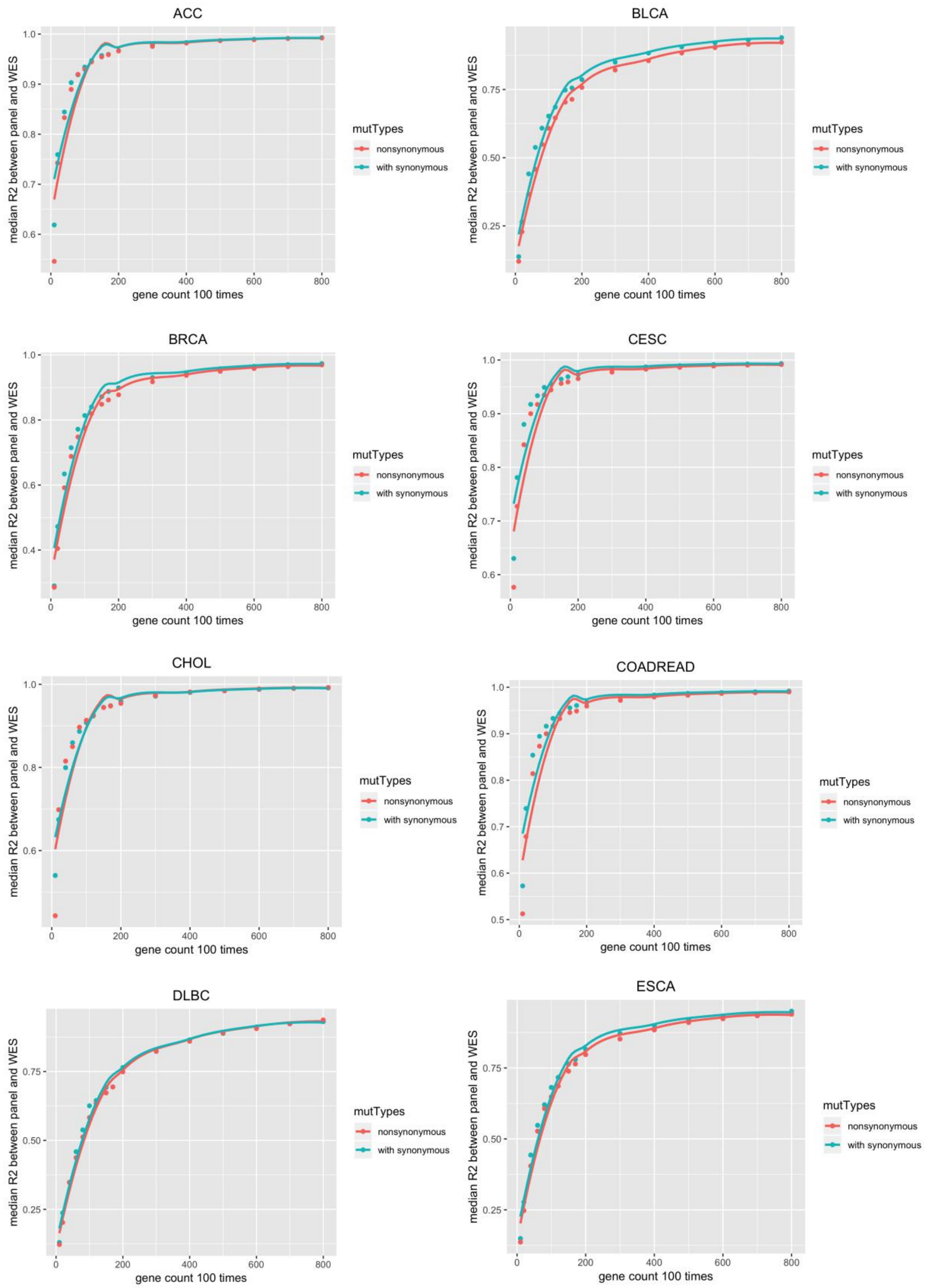

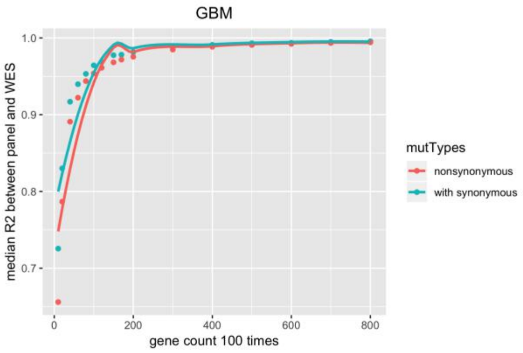
GBM
median R2 between panel and WES
gene count 100 times
mutTypes
nonsynonymous
with synonymous

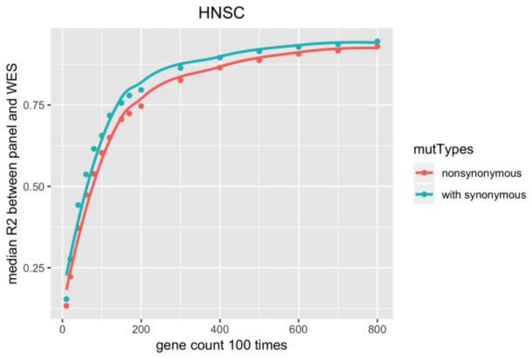
HNSC
median R2 between panel and WES
gene count 100 times
mutTypes
nonsynonymous
with synonymous

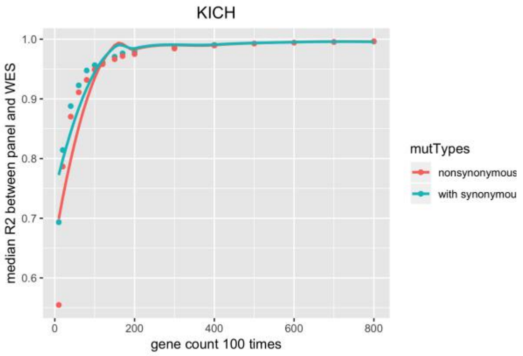
KICH
median R2 between panel and WES
gene count 100 times
mutTypes
nonsynonymous
with synonymou

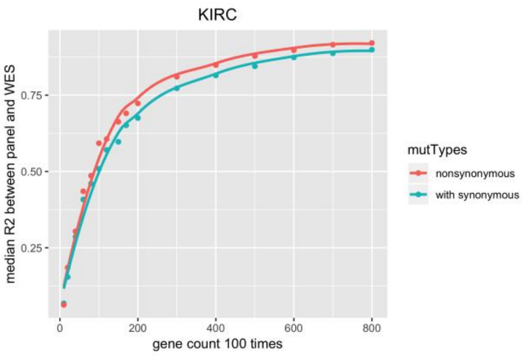
KIRC
median R2 between panel and WES
gene count 100 times
mutTypes
nonsynonymous
with synonymous

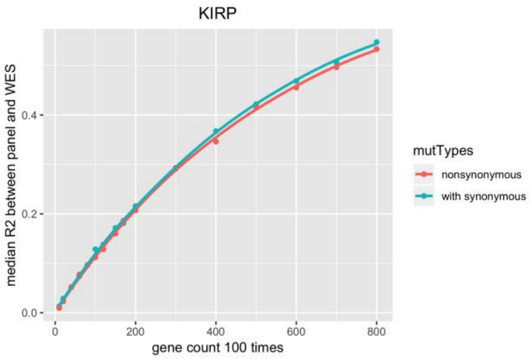
KIRP
median R2 between panel and WES
gene count 100 times
mutTypes
nonsynonymous
with synonymous

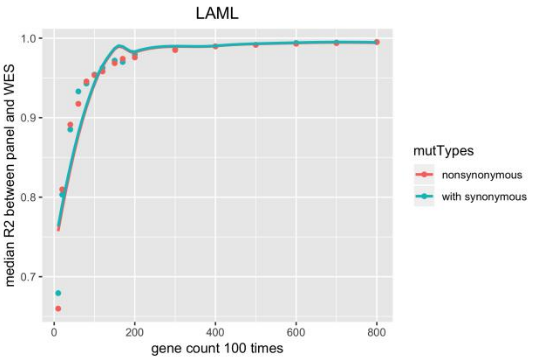
LAML
median R2 between panel and WES
gene count 100 times
mutTypes
nonsynonymous
with synonymous

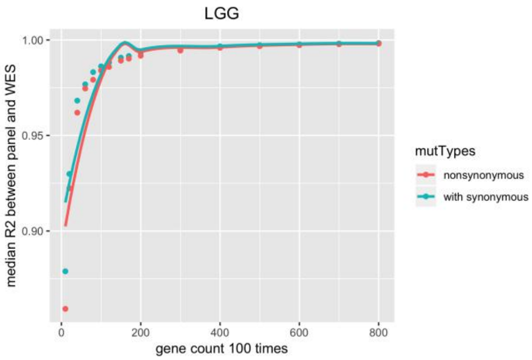
LGG
median R2 between panel and WES
gene count 100 times
mutTypes
nonsynonymous
with synonymous

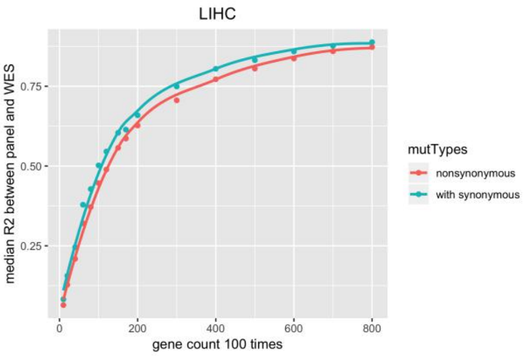
LIHC
median R2 between panel and WES
gene count 100 times
mutTypes
nonsynonymous
with synonymous

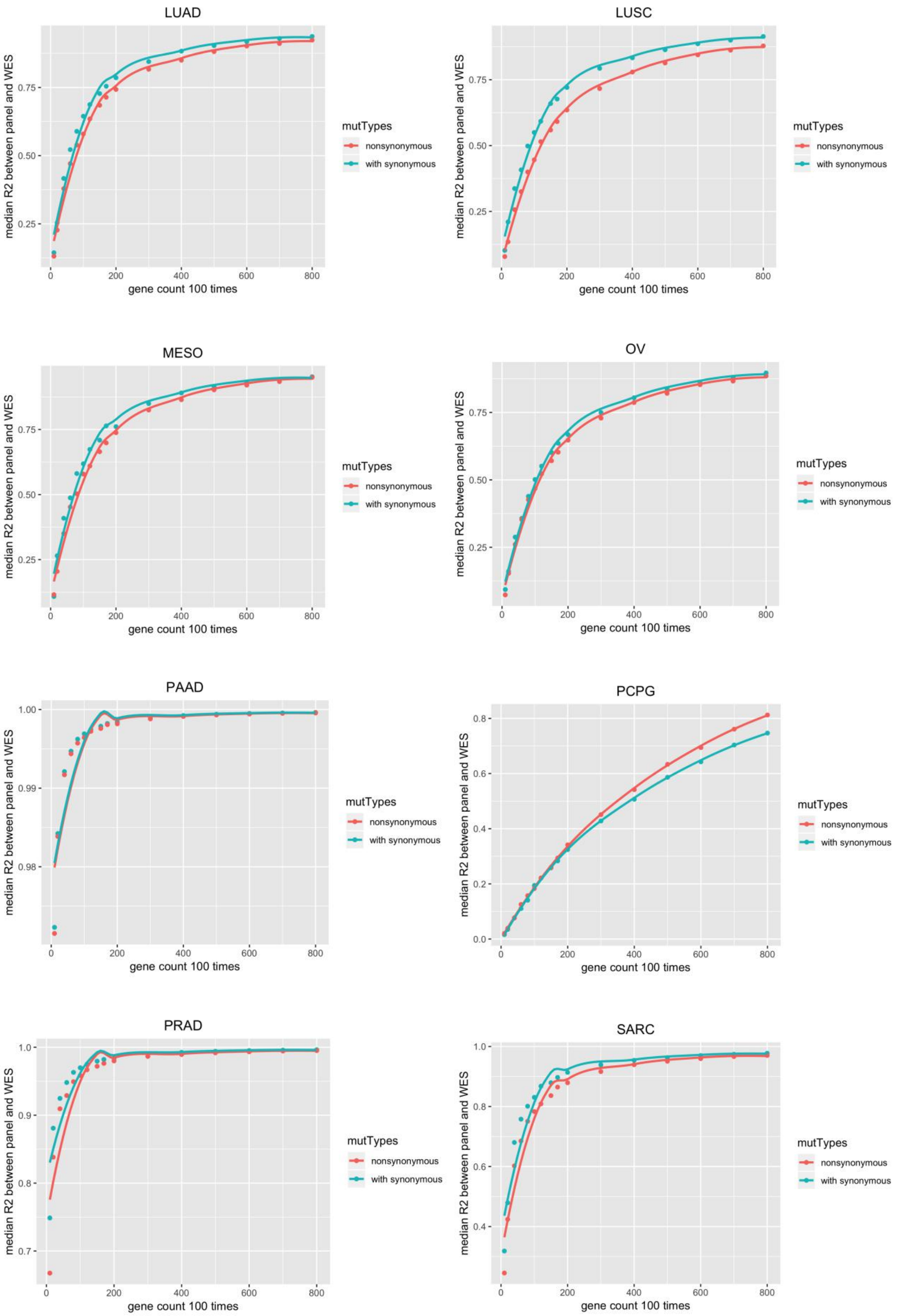
LUAD
LUSC
MESO
OV
PAAD
PCPG
PRAD
SARC
median R2 between panel and WES
gene count 100 times
mutTypes
nonsynonymous
with synonymous

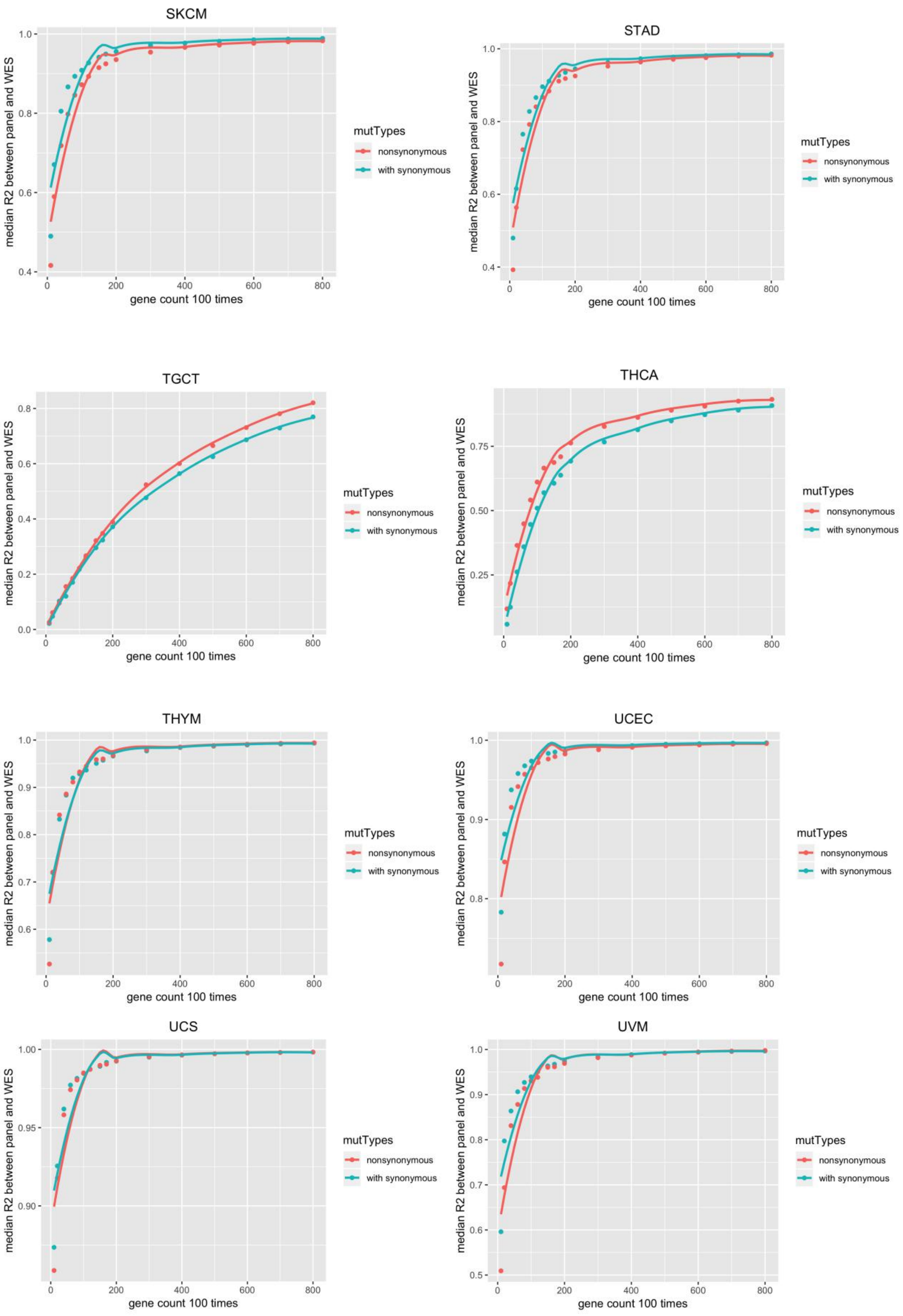
SKCM
STAD
TGCT
THCA
THYM
UCEC
UCS
UVM
median R2 between panel and WES
gene count 100 times
mutTypes
nonsynonymous
with synonymous

Figure S5. Driver gene count in SepPanel cross 32 cancer types. Orange bar represents the gene count of SepPanel, blue bar represents overlap of SepPanel with driver genes.

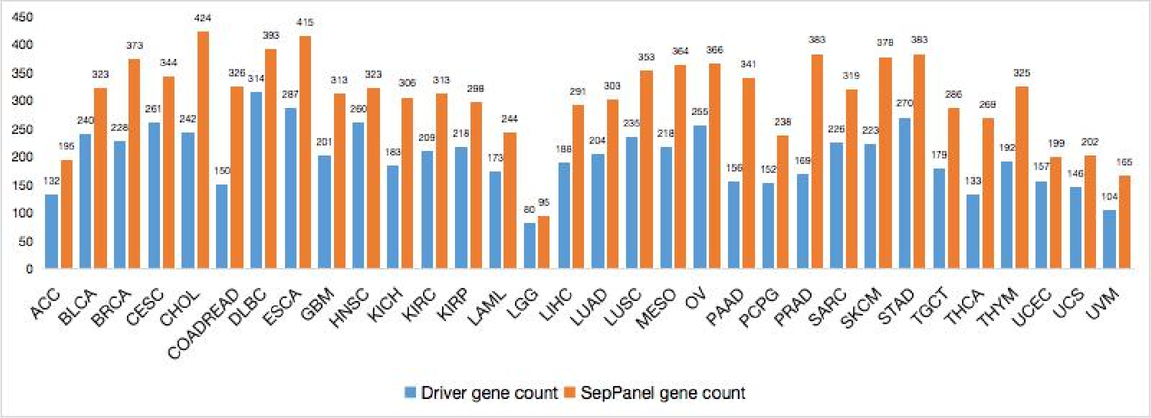

Table S1. Cancer types and the relative number of samples.

Cancer Types	Abbreviations	Sample counts
Acute Myeloid Leukemia	LAML	149
Adrenocortical carcinoma	ACC	92
Bladder Urothelial Carcinoma	BLCA	412
Brain Lower Grade Glioma	LGG	513
Breast invasive carcinoma	BRCA	1044
Cervical squamous cell carcinoma and endocervical adenocarcinoma	CESC	305
Cholangiocarcinoma	CHOL	51
Colon adenocarcinoma/Rectum adenocarcinoma Esophageal carcinoma	COADREAD	591
Esophageal carcinoma	ESCA	184
Glioblastoma multiforme	GBM	396
Head and Neck squamous cell carcinoma	HNSC	510
Kidney Chromophobe	KICH	66
Kidney renal clear cell carcinoma	KIRC	339
Kidney renal papillary cell carcinoma	KIRP	288
Liver hepatocellular carcinoma	LIHC	375
Lung adenocarcinoma	LUAD	569
Lung squamous cell carcinoma	LUSC	497
Lymphoid Neoplasm Diffuse Large B-cell Lymphoma	DLBC	48
Mesothelioma	MESO	83
Ovarian serous cystadenocarcinoma	OV	443
Pancreatic adenocarcinoma	PAAD	183
Pheochromocytoma and Paraganglioma	PCPG	179
Prostate adenocarcinoma	PRAD	498
Sarcoma	SARC	255
Skin Cutaneous Melanoma	SKCM	470
Stomach adenocarcinoma	STAD	441
Testicular Germ Cell Tumors	TGCT	150
Thymoma	THYM	123
Thyroid carcinoma	THCA	496
Uterine Carcinosarcoma	UCS	57
Uterine Corpus Endometrial Carcinoma	UCEC	542
Uveal Melanoma	UVM	80

Table S2. SepPanels in 32 cancer types.(Please see excel file in zip named “TableS2.xlsx”. The file “TableS2” is as a separate supplementary file in “supplementary_material.zip”, not included in this supplementary PDF.)

Table S3. Genes may derive neoantigens. Genes with upregulation of gene expression, high mutation frequency and high MHC affinity in CRC-, STAD-, and UCEC- SepPanel, F1CDx and MSK gene panel.

Genes	Resource	STAD-SepPanel	F1CDx	MSK	Cancer types
AXIN2	Rooney/TCIA/TSNAdb	√	√	√	CRC
BRCA2	Rooney/TSNAdb	√	√	√	CRC
COL5A3	Rooney/TCIA/TSNAdb		√		CRC
DNAH12	Rooney		√		CRC
DNAH12	TSNAdb		√		CRC
DNMT3B	Rooney/TCIA/TSNAdb			√	CRC
EPHB1	Rooney/TSNAdb		√	√	CRC
MUC16	Rooney/TSNAdb	√			CRC
ROBO2	Rooney/TSNAdb	√			CRC
ASPM	Rooney/TCIA/TSNAdb	√	√		STAD
BRCA2	Rooney/TCIA/TSNAdb	√	√	√	STAD
CACNA1D	Rooney/TCIA/TSNAdb	√			STAD
CARD11	Rooney/TCIA/TSNAdb	√	√	√	STAD
COL1A2	Rooney/TSNAdb	√			STAD
COL5A1	TCIA/TSNAdb	√	√		STAD
DMBT1	Rooney/TCIA/TSNAdb	√			STAD
EPHB2	Rooney/TCIA/TSNAdb	√			STAD
FN1	Rooney/TCIA/TSNAdb	√	√		STAD
MUC16	Rooney/TSNAdb	√			STAD
POLQ	Rooney/TCIA/TSNAdb	√			STAD
RNF43	Rooney/TCIA/TSNAdb	√		√	STAD
ROS1	Rooney/TSNAdb	√		√	STAD
SALL4	Rooney/TSNAdb	√			STAD
ALK	Rooney/TSNAdb		√	√	UCEC
ALPK2	Rooney/TSNAdb		√		UCEC
ANK1	Rooney/TSNAdb	√			UCEC
ASPM	Rooney/TCIA/TSNAdb	√	√		UCEC
BLM	Rooney/TCIA/TSNAdb		√	√	UCEC
BRCA1	Rooney/TCIA/TSNAdb	√	√	√	UCEC
BRCA2	Rooney/TSNAdb	√	√	√	UCEC
BRIP1	Rooney/TSNAdb		√	√	UCEC
CDH1	Rooney/TCIA/TSNAdb	√	√	√	UCEC
CHEK2	Rooney/TCIA/TSNAdb		√	√	UCEC

CIT	Rooney/TCIA/TSNAdb	√			UCEC
DNMT3B	Rooney/TCIA/TSNAdb			√	UCEC
ERBB3	Rooney/TCIA/TSNAdb		√	√	UCEC
ERBB4	Rooney/TSNAdb		√	√	UCEC
EZH2	Rooney/TCIA/TSNAdb		√	√	UCEC
FANCA	Rooney/TCIA/TSNAdb		√	√	UCEC
FANCD2	Rooney/TCIA/TSNAdb		√		UCEC
FAT2	Rooney/TSNAdb	√			UCEC
FBN2	Rooney/TCIA/TSNAdb		√		UCEC
MST1R	Rooney/TCIA/TSNAdb			√	UCEC
MUC16	Rooney/TCIA/TSNAdb	√			UCEC
MUC4	TCIA/TSNAdb	√			UCEC
MYH14	Rooney/TCIA/TSNAdb	√			UCEC
POLQ	Rooney/TSNAdb	√			UCEC
RNF43	Rooney/TCIA/TSNAdb	√		√	UCEC
